# Distribution of an Invasive Aquatic Pathogen (Viral Hemorrhagic Septicemia Virus) in the Great Lakes and Its Relationship to Shipping

**DOI:** 10.1371/journal.pone.0010156

**Published:** 2010-04-13

**Authors:** Mark B. Bain, Emily R. Cornwell, Kristine M. Hope, Geofrey E. Eckerlin, Rufina N. Casey, Geoffrey H. Groocock, Rodman G. Getchell, Paul R. Bowser, James R. Winton, William N. Batts, Allegra Cangelosi, James W. Casey

**Affiliations:** 1 Department of Natural Resources, Cornell University, Ithaca, New York, United States of America; 2 Department of Microbiology and Immunology, College of Veterinary Medicine, Cornell University, Ithaca, New York, United States of America; 3 Western Fisheries Research Center, U.S. Geological Survey, Seattle, Washington, United States of America; 4 Northeast-Midwest Institute, Washington, D. C., United States of America; University of Georgia, United States of America

## Abstract

Viral hemorrhagic septicemia virus (VHSV) is a rhabdovirus found in fish from oceans of the northern hemisphere and freshwaters of Europe. It has caused extensive losses of cultured and wild fish and has become established in the North American Great Lakes. Large die-offs of wild fish in the Great Lakes due to VHSV have alarmed the public and provoked government attention on the introduction and spread of aquatic animal pathogens in freshwaters. We investigated the relations between VHSV dispersion and shipping and boating activity in the Great Lakes by sampling fish and water at sites that were commercial shipping harbors, recreational boating centers, and open shorelines. Fish and water samples were individually analyzed for VHSV using quantitative reverse transcription-polymerase chain reaction (qRT-PCR) and cell culture assays. Of 1,221 fish of 17 species, 55 were VHSV positive with highly varied qRT-PCR titers (1 to 5,950,000 N gene copies). The detections of VHSV in fish and water samples were closely associated and the virus was detected in 21 of 30 sites sampled. The occurrence of VHSV was not related to type of site or shipping related invasion hotspots. Our results indicate that VHSV is widely dispersed in the Great Lakes and is both an enzootic and epizootic pathogen. We demonstrate that pathogen distribution information could be developed quickly and is clearly needed for aquatic ecosystem conservation, management of affected populations, and informed regulation of the worldwide trade of aquatic organisms.

## Introduction

Viral hemorrhagic septicemia virus (VHSV) is a rhabdovirus that has caused extensive losses of cultured and wild fish. It is one of the most studied fish pathogens [1] and has expanded its geographic range and habitat occupation in the last two decades. Up to the mid-1980s, VHSV was considered [2] to be a pathogen limited to freshwater fish in western Europe. VHSV was later detected [3], [4] in Pacific salmon (*Oncorhynchus tshawytscha*, *O. kisutch*) and then determined [1], [5], [6] to be present in many fish species of the North Pacific and North Atlantic oceans. A survey of a wide range of marine species in the North Sea [7] in the late 1990s found VHSV in many species examined even though few displayed clinical signs of the disease. VHSV now appears widely distributed in the Northern Hemisphere across many species that may or may not show symptoms of viral infection or experience mortality.

**Figure 1:**
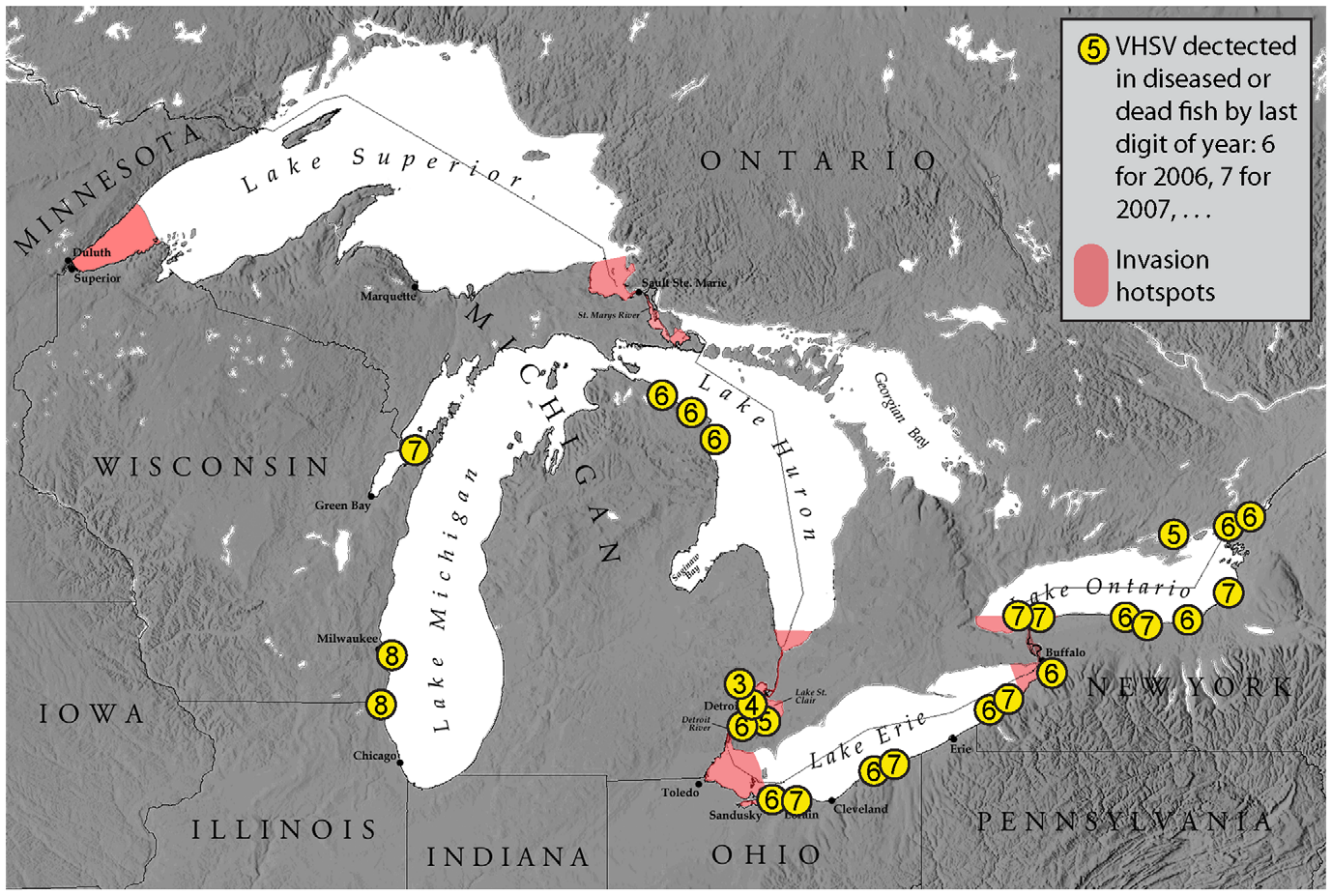
Distribution of VHSV positive fish in the Great Lakes from 2003 through 2008 as reported by the U.S. Department of Agriculture Animal and Plant Health Inspection Service [12] and the distribution of documented invasion hotspots [18], [27].

More recently, a novel genotype of VHSV (North American genotype IVb) has become established in the North American Great Lakes and is expanding to other freshwaters in the United States. The virus was first isolated in the Great Lakes in 2005 from freshwater drum (*Aplodinotus grunniens*) and round goby (*Neogobius melanostomus*) experiencing a large die-off in Lake Ontario [8]. Afterward, VHSV was isolated from archived samples of muskellunge (*Esox masquinongy*) captured in Lake St. Clair, Michigan in 2003 [9]. Additional fish mortality episodes appeared in 2006 through 2008 at several locations in Lakes Michigan, Erie, St. Clair, and connected waters ([9]–[12], Figure 1). Most investigations of VHSV in the Great Lakes have been associated with fish mortality in new areas of the system.

The process of VHSV range expansion is not understood. The emergence of large fish die-offs beginning in 2005 led management agencies and the public to regard VHSV as a recent invader. Introduction of VHSV into the Great Lakes via ship ballast water has been postulated as a likely mechanism [8], [13]–[15] because ballast water has been implicated in a majority of non-native species introductions in the Great Lakes [16]–[19]. Investigations of ship ballast contents further substantiates the likelihood that shipping is a key source of new aquatic species entering the Great lakes [20]–[22]. In addition, non-native bacteria and viruses have been documented in ship ballast at ports [23]–[25]. Shipping and recreational boating activity within the Great Lakes region have also been considered [26] as a mechanism for non-native species dispersion. As a result, controls on ship ballast water are being developed under intense public concern about new species entering the Great Lakes.

Fish kills caused by VHSV have elevated public and government attention on species introductions in the Great Lakes with attention focused on ships and ports for monitoring and regulation. Shallow connecting waters between the lakes and shallow approaches to primary shipping ports are considered invasion hotspots [18], [27] from the record of first occurrence of non-native species and expected discharge of ballast water in these areas (Figure 1). Ship decontamination and port monitoring are being targeted by conservation organizations, federal agencies, state governments, and others (e.g., [27]–[29]) to prevent invasions by non-native species, including microbes. However, there are currently no data to corroborate a primary role of ships in introduction or dispersal of VHSV. Instead, obvious fish die-offs have been the de facto method for detecting pathogen presence. This approach does little to inform options for prevention of pathogen dispersal by ships and other vectors.

Our purposes for this study were to: (1) test the capability to detect VHSV in Great Lakes water samples and fish using rapid survey methods suitable for regional-scale monitoring, and (2) to test the hypothesis that ports and invasion hotspots are points of concentration for VHSV.

## Results

Fish collections at the 30 sites yielded 1,221 fish of 17 species (Table 1) that were analyzed for VHSV. Most were round goby (710) and yellow perch (264) with median total lengths of 91 and 195 mm, respectively (Table 2). Six fish species were found to have VHSV and most positive fish were round goby and yellow perch. The qRT-PCR titers in the 55 positive fish were highly varied (1 to 5,950,000 N gene copies). The highest N gene count per fish was 48 times higher than the next highest so we considered this value an outlier. Based on the other 54 fish, N gene counts averaged 4,202 per sample (standard deviation 17,419) with a median count of 115 (interquartile range of 37 to 1,400). Four of the 55 qRT-PCR positive fish were confirmed VHSV positive by cell culture analyses (Table 1). These were the four fish with the highest N gene counts (>16,500). Highly variable N gene counts and the dominance of VHSV negative fish essentially resulted in a dichotomous data distribution: VHSV presence (detected) or absence (not detected) by fish. By site, the number of VHSV positive fish was highly correlated (r = 0.93, P = 0.0007) with the number of fish tested. With a mean detection rate of 5% (95% CI 0–14) half of the species were not collected in enough numbers to reliably detect VHSV. N gene counts in 9 positive 10 L water samples averaged 100 per sample (standard deviation 70) with an interquartile range of 48 to 160. Water sample results were also treated as presence or absent for consistency with fish analysis results.

**Table 1:** VHSV detection in fish and water using qRT-PCR and cell culture assays shown by site, type of site, and number of fish tested.

Site #	Body of water	Site Type	Invasion hotspot	Fish tested	VHSV positive fish	Cell culture positive fish	VHSV detected in water
1	St. Lawrence River	Recreational		60	0	0	
2	St. Lawrence River	Open shore		22	1	0	Yes
3	St. Lawrence River	Shipping		22	0	0	
4	Lake Ontario	Shipping		23	2	0	Yes
5	Lake Ontario	Recreational		60	2	0	Yes
6	Lake Ontario	Open shore		56	5	0	
7	Lake Erie	Shipping	Yes	21	1	0	
8	Lake Erie	Recreational		72	4	0	
9	Lake Erie	Open shore		36	1	0	Yes
10	Lake Erie	Recreational		21	0	0	
11	Lake Erie	Open shore		14	0	0	
12	Lake Erie	Shipping		103	3	0	
13	Lake Erie	Open shore	Yes	24	4	0	
14	Lake Erie	Recreational		23	1	0	Yes
15	Lake Erie	Open shore	Yes	35	1	0	Yes
16	Detroit River	Recreational	Yes	44	1	1	
17	Detroit River	Shipping	Yes	38	2	0	Yes
18	Lake Erie	Shipping	Yes	68	2	0	Yes
19	Lake Erie	Recreational		36	2	1	Yes
20	Lake Erie	Shipping		35	0	0	
21	Lake Erie	Open shore		36	0	0	
22	Lake Huron	Open shore		39	0	0	
23	Lake Huron	Recreational		63	0	0	
24	Lake Huron	Shipping		34	2	0	
25	St. Marys River	Shipping	Yes	42	0	0	
26	Lake Huron	Recreational		41	1	0	
27	Lake Huron	Open shore		21	7	2	
28	Lake Ontario	Recreational		66	3	0	
29	Lake Ontario	Shipping		39	2	0	
30	Lake Ontario	Open shore		27	8	0	

**Table 2:** Fish analyzed for VHSV and number of fish determined to be positive with data on fish sizes and known vulnerability to VHSV.

Common name	Scientific name	VHSV susceptible^1^	Number analyzed	VHSV positive	Median total length (mm)	Length range (mm)
Round goby	*Neogobius melanostomus*	Yes	710	41	91	45–230
Yellow Perch	*Perca flavescens*	Yes	264	6	195	150–240
Rock bass	*Ambloplites rupestris*	Yes	69	3	121	65–219
White perch	*Morone americana*	Yes	48	2	195	150–240
Banded killifish	*Fundulus diaphanus*	No	40	1	73	61–81
Spottail shiner	*Notropis hudsonius*	Yes	24	0	124	30–146
White sucker	*Catostomus commersonii*	No	22	0	225	211–263
White bass	*Morone chrysops*	Yes	15	2	182	157–198
Freshwater drum	*Aplodinotus grunniens*	Yes	7	0	164	135–187
Gizzard shad	*Dorosoma cepedianum*	Yes	6	0	169	155–204
Walleye	*Sander vitreus*	Yes	6	0	240	225–281
Bluegill	*Lepomis macrochirus*	Yes	3	0	141	54–164
Channel catfish	*Ictalurus punctatus*	Yes	2	0	261	260–263
Logperch	*Percina caprodes*	No	2	0	117	100–135
Pumpkinseed	*Lepomis gibbosus*	Yes	1	0	135	-
Largemouth bass	*Micropterus salmoides*	Yes	1	0	125	-
Golden shiner	*Notemigonus crysoleucas*	No	1	0	169	-

1. VHSV susceptibility is reported by the U.S. Department of Agriculture, Animal and Plant Health Inspection Service [12] although other species can be infected.

The presence of VHSV in fish and water samples from the same site were associated (Table 3, chi-square test, P = 0.0189). Matching positive and negative detections for fish and water were more common (9 sites) than expected assuming no association (6 sites). Also, VHSV was not detected in water samples at any site where VHSV was not detected in fish. The zero value for one cell in Table 3 violates an assumption of chi-square tests, but an alternative test for such counts (Fisher Exact Test) provided the same conclusion.

**Table 3:** Detection of VHSV in fish and water and the presence of VHSV positive fish at sampling sites in and outside of invasion hotspots shown in Figure 1.

Site attribute	VHSV present in fish	VHSV absent in fish
VHSV in water	9	0
VHSV absent in water	12	9
Invasion hotspot	6	1
Not an invasion hotspot	15	8

**Figure 2:**
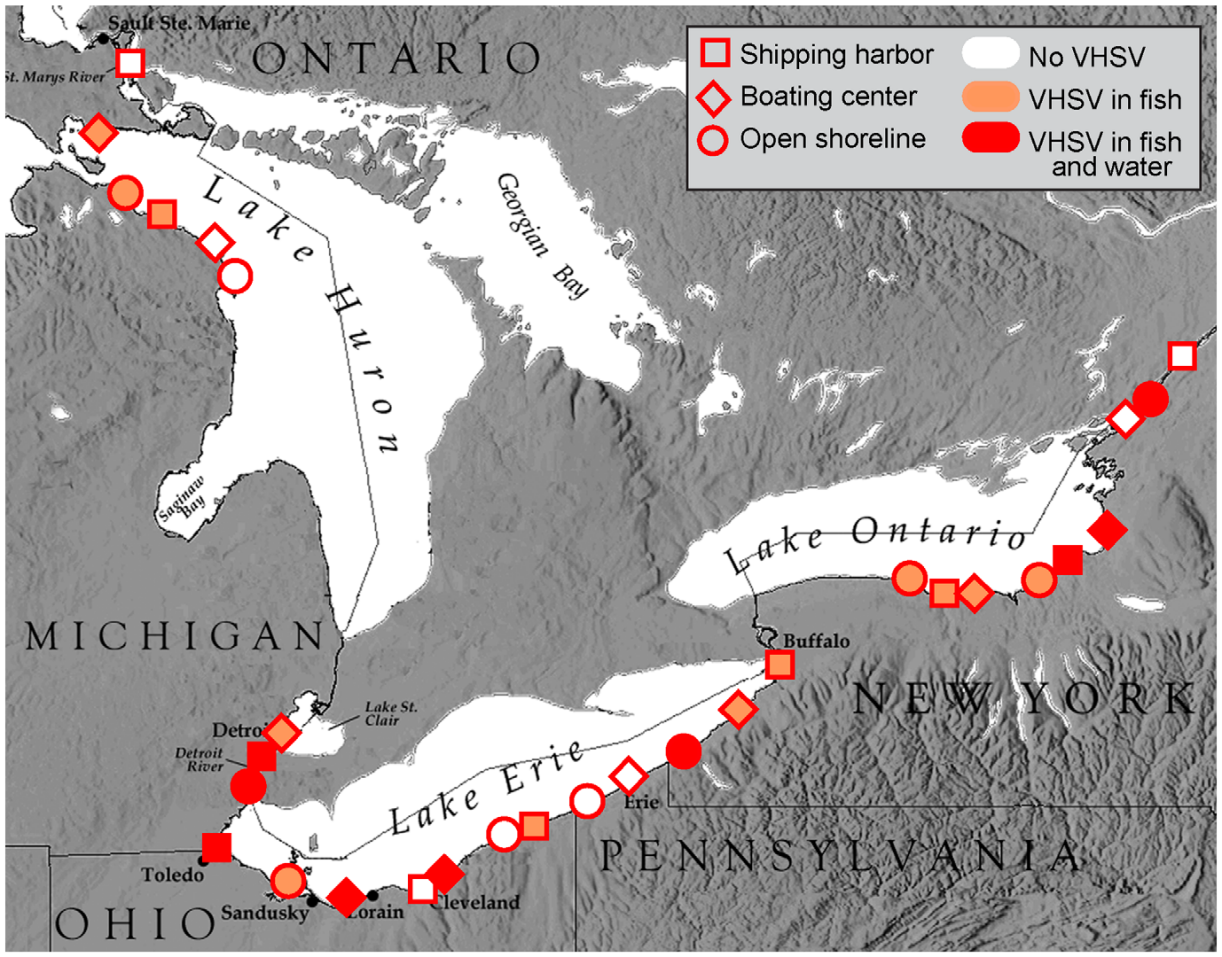
Distribution of VHSV positive fish and water at sites classified as commercial shipping harbors, recreational boating centers, and open shoreline.

Seven of the 30 sampling sites were in known invasion hotspots (Figure 1) [18], [27] of the Great Lakes. The distribution of VHSV positive sites was not associated with these hotspots (Table 3, chi-square test, P = 0.3001) and most of the VHSV positive sites (Figure 2) were not in invasion hotspots. The first detection of VHSV in the Great Lakes (2005 in northeast Lake Ontario, Figure 1) was not in an invasion hotspot. Afterward, positive test results using archived fish from 2003 were from a hotspot (Lake St. Clair, Figure 1). Most subsequent VHSV detections were outside hotspot areas. Together, our results and the timeline of VHSV occurrence in the Great Lakes provide strong evidence that VHSV distribution was unrelated to invasion hotspots in 2008.

VHSV occurrence in fish and water at the sampling sites showed no relation to sites classified as commercial shipping harbors, recreational boating centers, and open shorelines (Table 4). A chi-square test of association between VHSV presence and absence and site class yielded a P of 1.000 because the counts were perfectly independent for both fish and water samples. The distribution of VHSV across sample sites shows nearly the maximum distributional range with the appearance of an even spread across the system (Figure 2). Therefore, we obtained very strong evidence that shipping and boating sites are independent of VHS distribution in 2008.

**Table 4:** Detection of VHSV in fish and water at sampling sites classified as commercial shipping harbors, recreational boat centers, and open shorelines.

VHSV status	Commercial harbors	Recreational boating centers	Open shoreline
Absent in fish	3	3	3
Present in fish	7	7	7
Absent in water	7	7	7
Present in water	3	3	3

Tests for differences between VHSV presence and absence in samples using factors for lake, water temperature, and sampling date were not significant for fish (MANOVA, P = 0.8896) or water samples (P = 0.3800). Testing each attribute individually (i.e., water temperature differences among positive and negative samples) provided the same results using an ANOVA (P>0.51 for fish; P>0.08 for water). Therefore, we can conclude there is no evidence that VHSV detection was related to sample site attributes in the study period and region.

## Discussion

Our results indicate a broad distribution of VHSV across much of the Great Lakes system in 2008. Most sites sampled had VHSV infected fish and many had VHSV detected in water despite the lack of large outbreaks or mass mortalities. The distribution of VHSV outbreaks in prior years and our detection of subclinical infections throughout a broad geographical range indicate that VHSV is both an enzootic and epizootic pathogen in the Great Lakes. This pattern of persistence and intermittent effects on numerous fish has been recognized for VHSV in other freshwater and marine systems [1], [4], [30]. Our results and the record of VHSV in freshwater and marine habitats of Europe and North America raise the possibility that VHSV was present in the Great Lakes for perhaps an extended period of years where it may have persisted in a subclinical form before the increase in infection pressure or other factors triggered major fish die-offs.

Biological invasions from ballast water introductions are a major conservation and regulatory concern [13], [27], [31] and a leading cause of biotic homogenization, species extinctions, and ecosystem disruption [20], [21], [32]. While some detections of VHSV in the Great Lakes occurred in known invasion hotspots, most fish die-offs were not concentrated in these areas. Although our data do not address the way VHSV entered the Great Lakes, our results indicate no current relation to centers of shipping or boating activity nor invasion hotspots. VHSV outbreaks have been associated with high densities, strong recruitment, and stress in vulnerable species [1]. It appears that once VHSV is established in a region the virus will become widespread, hosted by fish without disease symptoms, and capable of persistence at low but detectable levels. Occasionally epizootics are seen and investigated by natural resource agencies and pathologists. These periodic, lethal event associated investigations makes a causal link to any one vector as a colonization cause difficult to assess. Advance knowledge of pathogen presence and distribution would provide a solid basis for judging threats and likely causes of epizootics.

While documenting the cause of fish die-offs commanded attention in the Great Lakes region, the more important benefits are establishing pathogen distribution, changes through time, and associations with epizootic outbreaks. Genomic technologies like qRT-PCR have been effective for learning about VHSV in Europe and North America and they provide the option for secure and fast results. We have also shown in a related study [33] that our qRT-PCR assay is 100 to 1000 more sensitive at detecting VHSV than cell culture, although cell culture can evaluate infectivity and gene variants. This study also demonstrates that pathogens like VHSV can be detected across a large region at a level of effort compatible with common environmental monitoring programs. Routine monitoring of fish for population trends [34] and contaminants [35] is already well established in the Great Lakes system. Adding pathogen surveillance would be practical, cost efficient, and a minor effort addition to these programs. Water could be used for monitoring although current technology makes pathogen detection less effective than sampling vulnerable organisms. Our results show that infected fish must be present locally to produce detectable levels of VHSV in water samples. In time, technology may allow efficient monitoring using water samples which are also widely collected in many forms of environmental surveillance in the Great Lakes and worldwide.

Genetic research on VHSV has indicated some consistency in the genotypes of the Great Lakes (IVb), and oceans around North America [5]. The distinct North American and European VHSV lineages were thought to have diverged 500 or more years ago [36], [37]. Our results and the genetic findings run counter to the public thinking on VHSV as a recent development brought on by worldwide shipping in the Great Lakes. However, present knowledge does not suggest a location from which VHSV was introduced into the Great Lakes. Pathogen monitoring can provide environmental agencies with information upon which to base management and regulatory decisions such as restrictions on transport of organisms, curtailment of water movement by ships, or imposition of ship and boat disinfection treatments. More knowledge of pathogens in the environment could have strongly affected the debate about VHSV. We demonstrate that this information can be developed and is clearly needed for aquatic ecosystem conservation, management of affected populations, and informed regulation of the worldwide unintended transfer of aquatic pathogens.

## Materials and Methods

### Ethics statement

All fish were handled in strict accordance with good animal practice as defined by the Cornell University Institutional Animal Care and Use Committee and all fish uses was approved under protocol number 2008-0045.

### Field Sampling

Environmental monitoring that includes field sampling is often designed to cover one site per day with some time allocated to travel. Our survey was designed to sample fish and water across the US coast of three Great Lakes (Ontario, Erie, Huron) and connecting waters during one six week period of fieldwork in the Spring of 2008 (1 May to 10 June). Fish and water sampling sites were selected around a set of 10 commercial shipping harbors from the St. Lawrence River to Sault Ste. Marie (MI). Commercial shipping harbors were identified using port classifications reported in reviews of transoceanic species introductions [19], [27]. The limited number of commercial shipping harbors provided us with few choices thus almost all were sampled. The same number of recreational boating centers and open shoreline sites were also included to make a total of 30 sampling sites. Ten recreational boating centers were identified using information from beach monitoring reports, recreation boating facilities, marinas, and waterfront tourism guides. Bathing beaches and marina facilities were commonly co-located making recreation sites locations of concentrated human activity and recreational boating. The third class of sites was open shoreline classified by coastal land use data (as in [38], [39]) assembled for the International Joint Commission and the US Army Corps of Engineers. Open shoreline sites had some public activity because access and boat launching were needed. Recreational boating centers and open shorelines were identified within a short drive (couple hours) of selected commercial shipping harbors; when multiple choices were available the final selection was random. Sampling sites were categorized as inside documented invasion hotspots or not using maps of these areas [18],[27].

Fish sampling was aimed at species known to be vulnerable to VHSV (e.g., round goby, yellow perch) [12] that are often abundant in shoreline waters of the study lakes, easy to capture, and small. Our sampling goal was 60 fish but the limitation of one day per site took precedent. Fish capture methods were minnow traplines, angling, and small mesh (2.5–5 cm) gill nets generally deployed simultaneously at each site. Captured fish were frozen whole in a mobile freezer and periodically delivered to the Aquatic Animal Health Laboratory at Cornell University. At each field sampling site, one water sample was collected in or near fish sampling locations. Water was pumped onto a research boat and into a 10 L carboy using a dedicated bilge pump. Sediments and suspended material in the sample was minimized by pumping from an intake tube pulled vertically through about 2 m of the water column at locations about 3.5 to 6 m deep. The 10 L of water collected at each site was shipped overnight to the Aquatic Animal Health Laboratory at Cornell University. Common site descriptive data were collected: sampled water depth, water transparency, conductivity, location coordinates, and water temperature.

At the start of the fieldwork, our boat and gear was free of organic material, disinfected, and dried for many months. After each field day aquatic plants, animals, and mud were removed from our boat, trailer, equipment and gear. All water was drained from the boat, motor, bilge, transom wells, as well as from equipment and gear. The boat, equipment and gear were disinfected with bleach solution (5%) with 10 minutes of contact time. The boat and gear were dried between sampling sites; extra sampling gear was used to allow drying and disinfection.

### Laboratory Analyses

Fish were thawed until pliable and tissue samples (spleen, liver, anterior and posterior kidney, heart) were collected aseptically, with sterile forceps and dissection scissors, disinfected scalpel blade handle and sterile scalpel blade for every individual fish. A small portion (<10 mg) from each tissue of a fish were pooled with 200 µL HBSS and stored at −80°C for RNA isolation. Remaining tissue from dissected organs of a single fish were pooled and stored at −80°C for cell culture. Disinfection of dissection surfaces occurred between each fish with an iodophore solution. Disinfection of the dissection room occurred between sites. VHSV-qRT-PCR positive samples were processed for virus isolation using EPC (epithelioma papulosum cyprini) cells [40]. Cell culture procedures followed the techniques of the American Fisheries Society [41].

Ten-liter water samples were filtered through a 0.22 um Tuffryn membrane (Pall Life Sciences) at 8 psi. The effluent was concentrated to 300 ml by tangential flow through a hollow fiber cartridge rated at 100,000 NMWC (GE Healthcare). The pressure was kept at 8 psi throughout the concentration process and a water temperature of 10°C. The remaining concentrate was pelleted at 25K RPM in a SW28 rotor for 2 hrs at 7°C. The resulting pellet was stored at −80°C.

Total RNA was isolated by a modification of the RNA Bee manufacturer's Tel-Test protocols. All samples and reagents were kept on ice or chilled to 10°C. The tissue sampled was combined with 100–200 ul of autoclaved 1×PBS in a sterile 1.5 ml microcentrifuge tube and homogenized using a sterile plastic pestle. Guanidine thiocyanate was added to the RNA Bee reagent at a concentration of 0.2 g/ml. One milliliter of this reagent mix was added to the homogenized tissue, followed immediately by 0.2 ml of chloroform. The tube was then inverted 20 times and left on ice for 10 minutes. After centrifugation (Eppendorf 5415D) at room temperature for 10 minutes at 13,000 rpm, the upper, aqueous layer was removed and added to 800 ul of isopropanol. The tube was vortexed briefly and then stored on ice for a minimum of 20 minutes or overnight. The sample was again centrifuged at room temperature for 10 minutes at 13,000 rpm and the supernatant decanted. One milliliter of 75% ethanol was added to the pellet. Following a final centrifugation at room temperature for 10 minutes at 13,000 rpm, ethanol was carefully removed with a pipette and the RNA pellet air dried. The pellets were resuspended using 100–200 ul of DEPC-treated water. The resuspended total RNA was then incubated for 10 minutes in a 65°C water bath. The concentration of each sample was determined using a Beckman DU-40 Spectrophotometer (Beckman-Coulter). In preparation for qRT-PCR, dilutions of the total RNA samples were prepared to load 40 ng of total RNA per well of a 96 well plate.

The primers and probe were designed to target the N gene of VHSV IVb MI03 isolate [4], [5], [8], [11], [33], [42], [43]. The 400 base pair conserved region of the N gene was selected for primer and probe design using Applied Biosystems software [37]: Forward- 5′- ACCTCATGGACATCGTCAAGG - 3′, Reverse- 5′ - CTCCCCAAGCTTCTTGGTGA - 3′, Probe- 5′ -/56-FAM CCCTGATGACGTGTTCCCTTCTGACC/36-TAMSp/- 3′. The assay was run on an Applied Biosystems-Prism model 7700 sequence detector (ABI, Foster City, California) and performed according to ABI using their TaqMan One-Step RT-PCR Master Mix reagents. VHSV N gene copy number standards were prepared and run exactly according to published methods [33] Template controls were not included for each plate to control for false positives. There were no false positives in any of the runs.

Fish testing positive by qRT-PCR for VHSV, regardless of VHSV N gene copy number, were subjected to confirmatory testing using a standard cell culture assay [41]. Tissue samples from qRT-PCR positive fish were shipped to the Western Fisheries Research Center (Seattle Washington) on dry ice. Samples were thawed, homogenized, supernatant diluted to 1∶40 and 1∶400. Inoculum (200 uL) was added to two PEG-treated wells and two untreated wells, plus 200 uL was added to another well of a separate plate (MEM-5-tris). In total 1000 uL of each dilution was applied to wells for detection and isolation. These were incubated at 15°C and observed for up to 18 days.

### Data Analysis

The number of the VHSV IVb N gene copies obtained from fish and water samples were summarized and then reduced to VHSV presence (detected) or absence (not detected) because most results were negative yielding a largely dichotomous data distribution. The numbers of sites where VHSV was present or absent were analyzed by chi-square statistics relative to fish and water, invasion hotspots, and site class (commercial shipping harbors, recreational boating centers, open shoreline). Using the data from VHSV detections in fish, differences in sample groups with the presence or absence of VHSV were analyzed for site attributes of location, date, and temperature using MANOVA and ANOVA.
